# Pharmacokinetics, metabolite profiling, safety, and tolerability of inhalation aerosol of 101BHG-D01, a novel, long-acting and selective muscarinic receptor antagonist, in healthy Chinese subjects

**DOI:** 10.3389/fphar.2022.1064364

**Published:** 2022-12-15

**Authors:** Huaye Gao, Jintong Li, Xiaoping Chen, Zhanguo Sun, Gang Cui, Minlu Cheng, Li Ding

**Affiliations:** ^1^ Department of Pharmaceutical Analysis, China Pharmaceutical University, Nanjing, China; ^2^ Drug Clinical Trial Research Center, China-Japan Friendship Hospital, Beijing, China; ^3^ Beijing Shuobai Pharmaceutical Technology Co., Ltd., Beijing, China; ^4^ Nanjing Jiening Pharmaceutical Technology Co., Ltd., Nanjing, China

**Keywords:** 101BHG-D01, chronic obstructive pulmonary disease, long-acting muscarinic antagonist, pharmacokinetics, metabolite, safety

## Abstract

101BHG-D01 is a novel, long-acting, selective muscarinic receptor antagonist for the treatment of chronic obstructive pulmonary disease (COPD). A single-site, randomized, double-blind, placebo-controlled and dose-escalation study of 101BHG-D01 inhalation aerosol was conducted to evaluate its pharmacokinetics, metabolite profiling, safety and tolerability following the single inhaled doses ranged from 20 to 900 μg in healthy Chinese subjects. After inhalation, 101BHG-D01 was absorbed rapidly into plasma with the time to maximum concentration about 5 min, and eliminated slowly with the terminal phase half-life about 30 h. The cumulative excretion rates of 101BHG-D01 in feces and urine were about 30% and 2%, respectively, which showed the study drug was mainly excreted in feces. The maximum drug concentration and area under the plasma concentration-time curve increased with dose escalation in the range of 20–600 μg, but their values increased out of proportion to the whole studied doses. The main metabolic pathways were loss of phenyl group and hydroxylation. No metabolite that presented at greater than 10 percent of total drug-related exposure was observed. 101BHG-D01 was safe and well tolerated after administration. The study results indicate that 101BHG-D01 is a good candidate for the treatment of COPD and enable further clinical development in subsequent studies in patients.

**Clinical Trial Registration:** http://www.chinadrugtrials.org.cn; Identifier: CTR20192058.

## 1 Introduction

Chronic obstructive pulmonary disease (COPD) is a common, treatable and preventable chronic respiratory disease (Xu et al., 2022), characterized by persistent airflow limitation and associated with an abnormal inflammatory response in the airways and lungs (da Silva et al., 2022). According to the World Health Organization, COPD is the third leading cause of death worldwide, with 65 million people suffering from it and over 4 million people dying each year globally (Alfahad et al., 2021). Bronchodilators are the mainstay of pharmacological treatment of COPD (Anzueto and Kaplan, 2020). Two classes of bronchodilators can be distinguished: β_2_-adrenoreceptor agonists and muscarinic antagonists (Kabir and Morshed, 2015; van Haarst et al., 2019; Calzetta et al., 2021), with long-acting bronchodilators considered to provide more convenient and effective symptom relief than short-acting bronchodilators (Quinn et al., 2018). Long-acting muscarinic antagonists (LAMAs) are recommended as the first-line maintenance bronchodilator therapy in patients with stable COPD without significant symptoms but who have a high risk of exacerbations and those without a history of exacerbation but with significant symptoms (Rhee et al., 2019). The most frequently prescribed LAMA is tiotropium that reduces airflow limitation, dynamic hyperinflation and COPD exacerbations, and improves patients’ quality of life (Tashkin et al., 2008; Yohannes et al., 2013). Tiotropium has similar affinity to the subtypes of muscarinic receptors, M1 to M3 (Barnes, 2000), and it exhibits pharmacological effects through inhibition of M3 receptor at the smooth muscle leading to bronchodilation (Donohue et al., 2002). However, its action on the muscarinic M2 receptor in the heart may increase the risk of adverse cardiovascular outcomes in patients (Oba et al., 2015), and a variety of studies suggested a significantly increased risk of acute urinary retention associated with tiotropium in patients with COPD (Loke and Singh, 2013). Given the high prevalence of COPD and the undesirable safety profiles of tiotropium, the development of additional options is clearly warranted.

**FIGURE 1:**
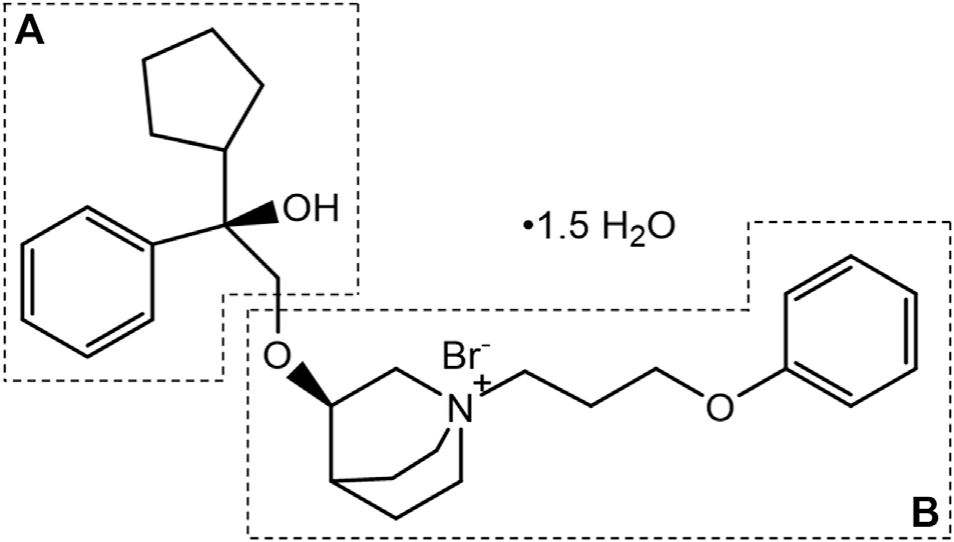
Chemical structure of 101BHG-D01.

101BHG-D01, chemically known as (R)-3-((R)-2-cyclopentyl-2-hydroxy-2-phenylethoxy)-1-(3-phenoxypropyl) quinuclidin-1-ium bromide sesquihydrate (Figure 1), is a novel, long-acting, selective muscarinic receptor antagonist for the treatment of COPD. The preparation method and medical use thereof have been patented in the United States (Patent No. US9751875B2). Compared with tiotropium, 101BHG-D01 has a greater affinity to M3 subtype and dissociates more rapidly from M2 subtype (Chu et al., 2022), resulting in a more preferable safety profile. Furthermore, 101BHG-D01 is considered to be administrated by inhalation and delivered *via* metered dose inhaler (MDI) due to its stable physical-chemical properties. Currently, 101BHG-D01 inhalation aerosol is undergoing Phase Ⅰb clinical trial in China, and it has shown the potential efficacy for the treatment of COPD.

Pharmacokinetics describes the fate of a drug in the body (i.e., the processes of absorption, distribution, metabolism, and excretion), which is necessary to evaluate the safety and effectiveness of the drug. Pharmacokinetic (PK) information is essential in establishing the most effective and safe therapeutic dose schedules for initiating and adjusting therapy in patient population. For instance, the half-life of a drug could help researchers to explain the accumulation of the drug in the body and optimize the frequency of administration. If a drug has a long half-life, the continuous administration could cause drug accumulation, which would raise safety concern, and the frequency of administration should be reduced. Meanwhile, metabolite profiling is strongly recommended by the U.S. Food and Drug Administration (FDA) to be conducted as early as feasible. Generally, metabolites identified only in human plasma or metabolites present at higher levels in humans than in any of the animal test species should be considered for safety assessment. If the human metabolites exceed 10 percent of total drug-related exposure at steady state, nonclinical pharmacodynamic and toxicity studies of the metabolites should be conducted. On the contrary, the above studies are not necessary temporarily. Therefore, we conducted this study to evaluate the pharmacokinetics, metabolite profiling, safety and tolerability of 101BHG-D01 following the single inhaled doses in healthy Chinese subjects.

## 2 Materials and methods

### 2.1 Chemicals and reagents

101BHG-D01 inhalation aerosols and placebo, as well as the reference substance of 101BHG-D01, were provided by Beijing Shuobai Pharmaceutical Technology Co., Ltd. (Beijing, China). The study drug was delivered *via* MDI, which containing 101BHG-D01 20 or 150 μg per actuation. The placebo formulation had no active substance, and the other substances were consistent with the corresponding 101BHG-D01 inhalation aerosol. Pooled human liver microsomes (HLMs, *n* = 50) were purchased from XenoTech, LLC (Lenexa, KS, United States). Nicotinamide adenine dinucleotide phosphate (NADPH) was obtained from Roche Diagnostic GmbH (Mannheim, Germany), and uridine diphosphate galacturonic acid (UDPGA) was purchased from Sigma-Aldrich (St. Louis, MO, United States). HPLC grade methanol and acetonitrile were from Merck KGaA (Darmstadt, Germany). All the other chemicals and reagents were of analytical grade and commercially available.

### 2.2 Study population

Subjects were male or female (non-lactating and non-pregnant) volunteers, 18–65 years old, with a body mass index (BMI) between 19–28 kg/m^2^, and assessed as healthy by review of medical history, physical examination, vital sign measurements, 12-lead electrocardiogram (ECG), and clinical laboratory evaluations. All subjects were non- or ex-smokers (<10 years and who had stopped smoking more than 3 months prior to screening) and had normal lung function. Subjects unable to tolerate the inhaled administration or with clinically significant medical conditions (based on the judgement of the investigators), including abnormal physical examination, laboratory tests and X-ray of the chest, or abnormal vital signs or ECG abnormalities were excluded. Other exclusion criteria were as follows: 1) a history of clinically diagnosed disease in circulatory, endocrine, nervous, digestive, urinary, respiratory, hematological, immunological system, malignant tumor, metabolic abnormality, or psychiatric disease; 2) a history of glaucoma, functional constipation, benign prostatic hyperplasia, urinary obstruction; 3) subjects with bronchitis, sinusitis, urinary tract infection, or cellulitis within 1 week prior to screening; 4) subjects with drug allergy history or allergic constitution; 5) subjects who received any medicine that inhibited or induced drug metabolic enzyme CYP2D6 or CYP3A4 (the study drug is a substrate for CYP2D6 and CYP3A4), or received systemic corticosteroid therapy and/or antibiotic therapy within 30 days before screening; 6) subjects who received any other medicine within 14 days preceding screening; 7) subjects who participated in another clinical trial with an investigational product within about 3 months prior to screening; 8) subjects with positive findings for human immunodeficiency virus, *Treponema pallidum* antibody, hepatitis B virus surface antigen, or anti-hepatitis C virus antibody; 9) a positive alcohol breathalyzer or urine screen for abused drugs and nicotine; (10) blood donation/loss more than 400 mL during the 3 months preceding screening. Written informed consents were obtained from all of the eligible subjects before the initiation of any study-related procedures.

### 2.3 Clinical study design

This was a single-site, randomized, double-blind, placebo-controlled and dose-escalation study consisting of six dose cohorts (20, 60, 150, 300, 600, and 900 μg) to evaluate the pharmacokinetics, metabolite profiling, safety and tolerability of 101BHG-D01 after inhalation in healthy Chinese subjects. Dose escalation was continued sequentially after review of all safety data and each subject could participate in only one dose cohort. In the 20 μg dose cohort, four subjects all received 101BHG-D01. In the 60–900 μg dose cohorts, the ten subjects of each cohort were randomized in a 4:1 ratio such that eight subjects received 101BHG-D01 inhalation aerosol and two subjects received the placebo. The sample size was determined to provide adequate PK, safety and tolerability data at each dose. For the allocation of the subjects, the randomization list was generated by using SAS statistical package version 9.4 (SAS Research and Development Co., Ltd., Chapel Hill, NC, United States). In this study, the study drug and placebo were identical in appearance to ensure both the investigators and the subjects were blind. The drug administration was conducted in accordance with the standard operating procedure (SOP) strictly. Prior to administration, all subjects received adequate administration training. The SOPs were as follows: 1) before using, remove the cap from the mouthpiece, shake the inhaler well and discard the first 2 sprays; 2) breathe out as fully as possible for 5 s, put the inhaler into mouth, and then close lips, keeping tongue below the mouthpiece; 3) while breathing in deeply and slowly for 4 s, press down on the center of the inhaler until the actuator stops moving; 4) after finishing breathing in, hold breath for 10 s and then breathe out gently for 5 s; 5) after using, rinse mouth with water to remove any excess medicine and do not swallow. As the clinical study was in progress, the metabolite profiling of 101BHG-D01 in human liver microsomes was conducted. According to the results of metabolite profiling *in vitro* (see the Section 3.3 “Metabolite profiling in human liver microsomes”), the main metabolite of 101BHG-D01was identified as M6, which corresponding to the loss of phenyl group compared with the parent drug. The pharmacokinetic profiles of M6 were also studied in the 600 and 900 μg dose cohorts. This study was carried out in accordance with the principles of the Declaration of Helsinki and the International Conference for Harmonization (ICH) E6 guidelines for Good Clinical Practice (GCP). The study protocol and informed consent form (ICF) were approved by the Clinical Research Ethics Committee of China-Japan Friendship Hospital (Beijing, China).

### 2.4 Sample collection and processing

In the 20–300 μg dose cohorts, blood samples were taken for pharmacokinetic analysis of 101BHG-D01. In the 600 and 900 μg dose cohorts, blood, urine and fecal samples were collected for the pharmacokinetic evaluation of 101BHG-D01 and M6, and estimation of the relative quantification of the parent drug and its metabolites.

In the 20 and 60 μg dose cohorts, blood samples were taken before dosing and at 5, 15, 30 min and 1, 2, 3, 4, 6, 8, 12, 24, 36, and 48 h after dosing. In the 150–900 μg dose cohorts, blood samples were taken before dosing and at 5, 15, 30 min and 1, 2, 4, 8, 12, 24, 36, 48, 72, and 96 h after dosing. Plasma was separated and stored at −60 to −90°C until analysis. Urine samples were collected within 24 h pre-dosing and the following time intervals: 0–2, 2–4, 4–8, 8–12, 12–24, 24–48, 48–72 and 72–96 h. The total volume of the urine in each time interval was recorded. The urine samples were stored at −60 to −90°C until analysis. Fecal samples were collected within the following time intervals: 24 h before dosing and 0–24, 24–48, 48–72 and 72–96 h. The fecal samples in each time interval were weighed. The fecal samples were stored at −20°C until analysis.

### 2.5 Pharmacokinetic assessments

Determination of 101BHG-D01 and M6 in plasma, urine and feces was performed by high performance liquid chromatography tandem mass spectrometry method (HPLC-MS/MS) using multiple reaction monitoring (MRM) detection. HPLC was performed with Shimadzu LC30AD (Shimadzu, Kyoto, Japan). Mass spectrometric detection was performed with Triple Quad 6500 (AB Sciex, Foster, United States) for plasma and urine, and QTrap5500 (AB Sciex, Foster, United States) for feces. These assays were all fully validated. The validated ranges were 1.00–800 pg/mL for 101BHG-D01 and 1.00–20.0 pg/mL for M6 in plasma, 0.0500–20.0 ng/mL for 101BHG-D01 and M6 in urine, 0.400–400 ng/mL for 101BHG-D01 and 0.100–100 ng/mL for M6 in feces.

The data acquisition was performed by AB Sciex Analyst software (version 1.6.3, AB Sciex, Foster, United States), and the data analysis was supported by Watson LIMS software (version 7.5, Thermo Fisher Scientific, Waltham, MA, United States). The pharmacokinetic parameters were calculated with WinNonlin version 8.0 (Pharsight Corporation, Mountain View, California) using non-compartmental analysis method. The main pharmacokinetic parameters of 101BHG-D01 and M6 in the study included the following: maximum concentration in plasma (C_max_); time to maximum concentration (T_max_); area under the concentration-time curve from time zero to the last measurable concentration (AUC_0-t_) or infinity (AUC_0-∞_); terminal phase half-life (t_1/2z_); renal clearance (CLr); the cumulative excretion rates of 101BHG-D01 in urine (fe_u_%) and feces (fe_f_%).

Statistical analysis was performed by using SAS statistical package version 9.4 (SAS Research and Development Co., Ltd., Chapel Hill, NC, United States). The relationship between the dose and systemic exposure to 101BHG-D01 was assessed using the method combining the log-linearized power model and the confidence interval (CI) criterion (Hummel et al., 2009). The model can be described as: $PK=\alpha \times {dose}^{\beta }$ . Linear regression of ln-transformed PK parameters by the ln-transformed dose was described in the form of $\ln\left(PK\right)=\ln\left(\alpha \right)+\beta \times \ln\left(dose\right)$ , where β was the dose proportionality coefficient (Hummel et al., 2009). Dose proportionality would be concluded if the 90% CI for β was within the acceptance range [ $1+\ln\left(0.8\right)/\ln\left(r\right)$ ; $1+\ln\left(1.25\right)/\ln\left(r\right)$ ], where r was the ratio of the highest to the lowest doses (Luo et al., 2021).

### 2.6 Metabolite profiling in human liver microsomes

The metabolite profiling of 101BHG-D01 in human liver microsomes was investigated by *in vitro* incubation with HLMs. The incubation mixture, in 0.1 M phosphate buffer (pH 7.4), consisted of 101BHG-D01 (1 μM), UDPGA (1 mM), NADPH (1 mM) and pooled human liver microsomes (1 mg/mL). The total incubation volume was 200 μL. After pre-incubation for 3 min at 37°C, the human liver microsomes were added to initiate the 1 h reaction at 37°C, which was then terminated with ice-cold acetonitrile. The negative control samples were prepared as described above with the addition of inactivated HLMs.

By using high performance liquid chromatography-quadrupole-time of flight mass spectrometry (HPLC-Q-TOF/MS) method, the metabolite profiling of 101BHG-D01 in human liver microsomes was conducted. The analysis platform was established by using a Shimadzu LC30AD HPLC system (Kyoto, Japan) followed a X500B QTOF (AB Sciex, CA, United States). The strategy was mainly divided into the following steps. The first step was on-line data acquisition. The OS software (version 1.6.1, AB Sciex, CA, United States) was applied for the data acquisition. A full mass scan was performed and the accurate MS/MS data sets were obtained using multiple mass defect filtering (MMDF)-dynamic background subtraction (DBS)-dependent data acquisition method. The application of MMDF and DBS could enable the mass spectrometry to distinguish between the background interfering ions and metabolite ions effectively and intelligently, and simultaneously gain the molecular ion peaks and MS/MS spectrum of the metabolites (Zhang et al., 2018). The second step was post acquisition data mining. Full scan mass spectra corresponding to the incubation and negative control samples were compared to look for potential metabolites. Only those analytes just presenting in the incubation samples were considered as potential metabolites. The extracted ion chromatograms (XIC) and MS/MS spectra of the possible components were investigated. Based on the qualitative information, such as the retention time (t_R_) and fragmentation ions, the metabolites were identified.

### 2.7 Relative quantification of 101BHG-D01 and metabolites in human plasma, urine and feces

Because of the low dose and poor systemic exposure after inhalation of 101BHG-D01, the concentrations of the parent drug and the metabolites *in vivo* was too low to be detected in the full mass scan by using HPLC-Q/TOF-MS method. Hence, the relative quantification of 101BHG-D01 and the metabolites in human plasma, urine and feces was conducted by using the LC-MS/MS method. The LC–MS/MS system consisted of the LC30AD liquid chromatographic system (Shimadzu, Kyoto, Japan) and Triple Quad 6500 mass spectrometer (AB Sciex, CA, United States). For the semi-quantitative profiling in circulation, the trapezoidal rule was used to yield the pooled samples that had the concentration proportional to the AUC (Hamilton et al., 1981; Hop et al., 1998) for each subject. For the relative quantification in urine and feces, sample pooling was conducted in proportion to the amount (volume or weight) of excreta collected in each sampling period (Penner et al., 2009). The proportion of 101BHG-D01 and each metabolite in these pooled samples was determined by the ratio of their peak area to the total peak area to represent their relative amounts.

Because of the extremely low concentration levels of 101BHG-D01 and M6 in the plasma samples collected from 24 to 96 h post-dosing, and to avoid unnecessary dilutions, the plasma samples ranged from 0 to 12 h post-dosing in the 600 and 900 μg dose cohorts were used to prepare the AUC pools for each subject (except for the placebo group). The urine and fecal samples collected up to 96 h post-dosing for each subject were mixed at the ratios according to all the collected sample volumes and weights, respectively.

The incubation, pre-dose and dose samples were analyzed sequentially. The ion pairs of 101BHG-D01 and the proposed metabolites were detected by using MRM with the same MS parameters. The ion pairs were chosen according to the MS/MS spectra of metabolite profiling in human liver microsomes. By comparing the retention time of each peak in XIC of the dosing samples with that of the incubation samples, the peaks of 101BHG-D01 and the metabolites in the dosing samples were identified. The blank samples were used as the negative controls.

### 2.8 Safety and tolerability assessments

During the study, all subjects remained in the clinical site under continuous observation. Safety signal monitoring included adverse events (AEs), serious adverse events (SAEs), physical examination, vital sign, pupillary measurement, laboratory examination (hematology, blood biochemistry, urinalysis, coagulation function) and ECG. AEs were graded using the Common Terminology Criteria for Adverse Events version 5.0 (CTCAE v5.0) (U.S. Department of Health and Human Services, 2017). Anticholinergic effects such as dry mouth, pupillary changes and tachycardia were specially focused. For the tolerability assessment, the next dose cohort would be tested when the investigators confirmed that the current dose was safe and well tolerated. In one dose cohort, if there was single drug-related SAE in the subjects receiving the study drug, the dose escalation was discontinued. If the drug-related AEs were reported as grade ≥2 (U.S. Department of Health and Human Services, 2017) in one-third or more of the subjects, the dose escalation was discontinued. If one-quarter or more of the subjects experienced the drug-related AEs of grade ≥3, or there were two same drug-related AEs of grade ≥3 (U.S. Department of Health and Human Services, 2017), the dose escalation was discontinued. When the dose escalation was discontinued, the former dose would be regarded as the maximum tolerated dose (MTD). After the study of 900 μg dose cohort was completed, the dose escalation was discontinued regardless of whether the subjects experienced the drug-related adverse events.

## 3 Results

### 3.1 Study population

A total of 250 Chinese volunteers were screened and 54 volunteers were enrolled in the study. The first subject was enrolled on 21 October 2019, and the last subject completed the follow-up on 17 May 2021. All the subjects completed the study according to the protocol. The baseline demographic data of the study population was summarized in Table 1.

**TABLE 1:** Baseline demographic data of the study population.

Characteristic	20 μg	60 μg	150 μg	300 μg	600 μg	900 μg	Placebo
	(*n* = 4)	(*n* = 8)	(*n* = 8)	(*n* = 8)	(*n* = 8)	(*n* = 8)	(*n* = 10)
Sex, n							
Male/female	2/2	1/7	4/4	3/5	4/4	4/4	3/7
Age, years							
Mean (SD)	31.5 (2.38)	33.0 (5.95)	32.6 (2.62)	33.4 (6.97)	35.4 (5.90)	29.4 (7.48)	33.6 (4.27)
Min-Max	29–34	23–41	29–37	21–42	25–42	21–42	26–39
Height, cm							
Mean (SD)	163.25 (11.663)	162.45 (5.967)	167.31 (8.101)	166.61 (10.992)	164.30 (5.725)	165.31 (9.859)	163.48 (6.271)
Min-Max	150–173.3	155.1–172.4	157.1–181.5	150.4–188.4	152.6–169.6	154.5–179	156.6–172.6
Weight, kg							
Mean (SD)	68.300 (9.3453)	62.763 (7.8747)	61.719 (8.2330)	64.638 (11.6430)	62.606 (6.7663)	62.231 (9.2449)	64.290 (8.0377)
Min-Max	56.55–77.3	53–76.2	53.95–77.65	45.45–83.95	51.9–71	47.5–75.7	52.7–79.2
BMI, kg/m^2^							
Mean (SD)	25.53 (0.750)	23.75 (2.282)	21.98 (1.326)	23.10 (1.838)	23.14 (1.354)	22.65 (1.493)	23.97 (1.584)
Min-Max	24.8–26.5	19.4–26.4	20–23.6	20.1–25.5	21.2–24.7	19.7–24.9	21–26.6

BMI, body mass index; SD, standard deviation; Max, maximum; Min, minimum.

### 3.2 Pharmacokinetic profiles

**FIGURE 2:**
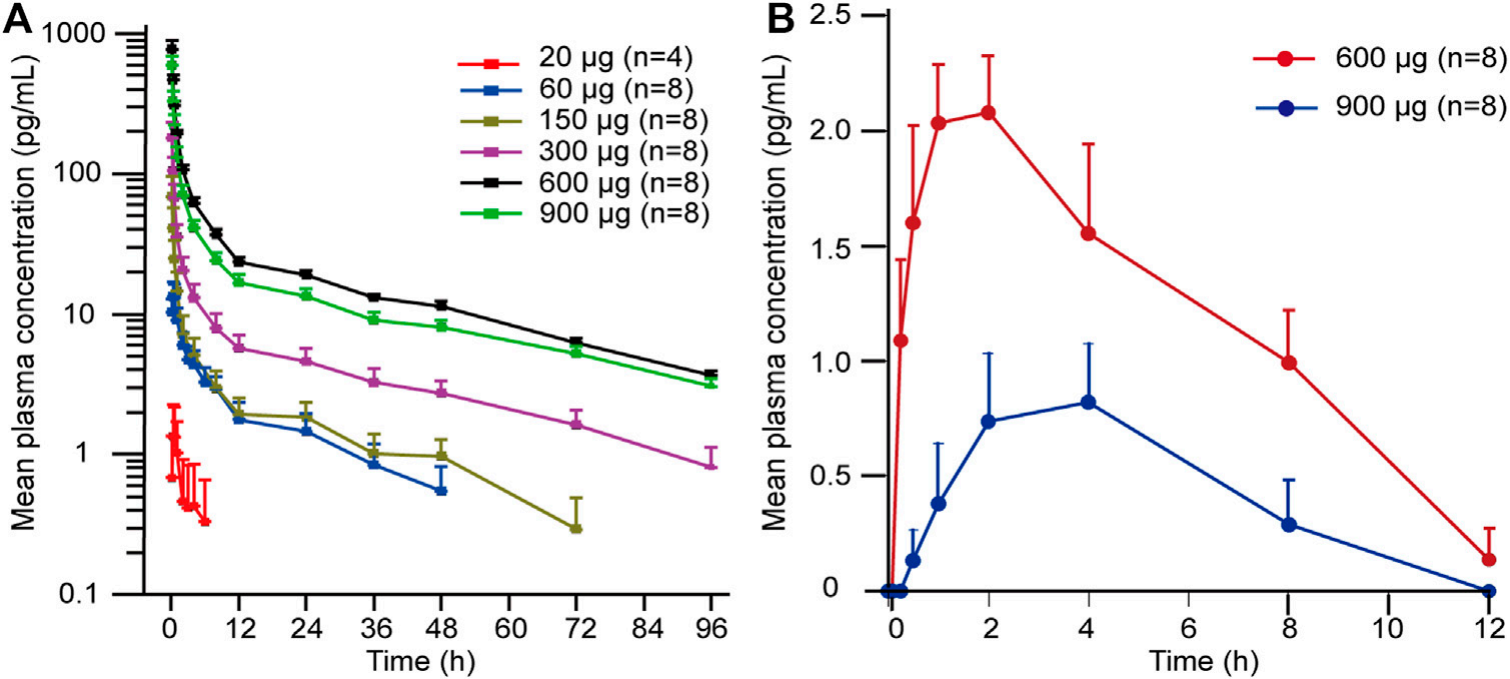
Mean plasma concentration-time curves of 101BHG-D01 and M6 after single inhaled doses of 101BHG-D01 inhalation aerosol in healthy Chinese subjects. **(A)** 101BHG-D01 (semi-logarithmic scale). **(B)** M6 (linear scale). Bars represent SDs. n, number of subjects.

The detailed main pharmacokinetic parameters of 101BHG-D01 and M6 are presented in Table 2. The mean plasma concentration–time curves of 101BHG-D01 and M6 are shown in Figure 2. 101BHG-D01 was rapidly absorbed into plasma with the median time to reach the peak concentration about 5 min after single dose inhalation. 101BHG-D01 was eliminated slowly with the mean values of t_1/2z_ about 30 h. M6, the main metabolite of 101BHG-D01, reached its peak concentration later than the parent drug, with the median time about 1.5–2 h. The mean t_1/2z_ values of M6 about 7–8 h showed the metabolite eliminated more rapidly than the parent drug. The CLr values of 101BHG-D01 and M6 in the 600 μg dose cohort were 6821 ± 1525 mL/h and 11799 ± 4512 mL/h, respectively. The maximum drug concentration and area under the plasma concentration-time curve of 101BHG-D01 increased with dose escalation in the range of 20 μg–600 μg. For the dose proportionality analysis, the 20 μg dose cohort was excluded due to the lack of the subjects whose significant PK parameters could be calculated. Based on the log-linearized power model, the β values of the C_max_, AUC_0-t_ and AUC_0-∞_ over the dose range of 60–900 μg were 1.58, 1.355, 1.116, respectively. The 90% CIs of the C_max_, AUC_0-t_ and AUC_0-∞_ were (1.306, 1.855), (1.01, 1.7), and (0.796, 1.435), respectively, which were not fully contained in the pre-defined CI criterion (0.918, 1.082). Hence, the linear pharmacokinetic characteristic of 101BHG-D01 was not observed.

**TABLE 2:** Main pharmacokinetic parameters of 101BHG-D01 and M6 after single inhaled doses of 101BHG-D01 in healthy Chinese subjects.

Pharmacokinetic parameters	101BHG-D01						M6	
	20 μg	60 μg	150 μg	300 μg	600 μg	900 μg	600 μg	900 μg
n	4	8	8	8	8	8	8	8
C_max_, pg/mL	2.77 ± 1.64	13.83 ± 9.39	79.22 ± 76.98	181.1 ± 143.3	773.1 ± 317.8	594.8 ± 255.2	2.25 ± 0.75	1.46 ± 0.38
T_max_, min	22.8 (15–30)	16.2 (5–30)	5 (5–5)	5 (5–5)	5 (5–5)	5 (5–5)	90 (30–240)	120 (120–240)
AUC_0-t_, pg·h/mL	6.63 ± 7.70	96.69 ± 73.26	158.8 ± 130.6	423.4 ± 293.2	1884.0 ± 356.6	1337.8 ± 520.9	12.37 ± 7.87	5.79 ± 4.72
AUC_0-∞_, pg·h/mL	28.86^a^	150.9 ± 82.5	219.1 ± 151.0	494.4 ± 318.3	2039.7 ± 365.5	1491.1 ± 546.7	29.54 ± 7.80	NA
t_1/2z_, h	8.81^a^	21.53 ± 5.36	36.62 ± 41.60	32.76 ± 14.11	29.24 ± 2.71	34.77 ± 5.08	7.55 ± 1.66	NA
CLr, mL/h	NA	NA	NA	NA	6821 ± 1525	8038 ± 1884	11799 ± 4512	NA
fe_u_, %	NA	NA	NA	NA	2.36 ± 0.80	1.37 ± 0.69	0.05 ± 0.01	0.06 ± 0.04
fe_f_, %	NA	NA	NA	NA	32.54 ± 11.30	29.85 ± 13.37	0.80 ± 0.27	0.31 ± 0.06

C_max_, maximum concentration in plasma; T_max_, time to maximum concentration; AUC_0-t_, area under the concentration-time curve from time zero to the last measurable concentration; AUC_0-∞_, area under the concentration-time curve from time zero to infinity; t_1/2z_, terminal phase half-life; CLr, renal clearance; fe_u_%, the cumulative excretion rate in urine; fe_f_%, the cumulative excretion rate in feces; NA, not applicable.

All values are expressed as mean ± SD, except for T_max_ values, which are expressed as median (range).

^a^ In the 20 μg dose cohort, the AUC_0-∞_ value of only one subject could be calculated.

After single inhaled dose of 600 and 900 μg of 101BHG-D01, its cumulative excretion rates in urine were 2.36% and 1.37%, and its cumulative excretion rates in feces over the time intervals of 0–96 h were 32.54% and 29.85%, respectively. These data suggested that fecal excretion might be the major excretion pathway of the unchanged 101BHG-D01. The total cumulative excretion rates in urine and feces of M6 over the time intervals of 0–96 h of 600 and 900 μg were 0.85% and 0.37%, respectively, which showed the amounts of this metabolite in urine and feces were low.

### 3.3 Metabolite profiling in human liver microsomes

**FIGURE 3:**
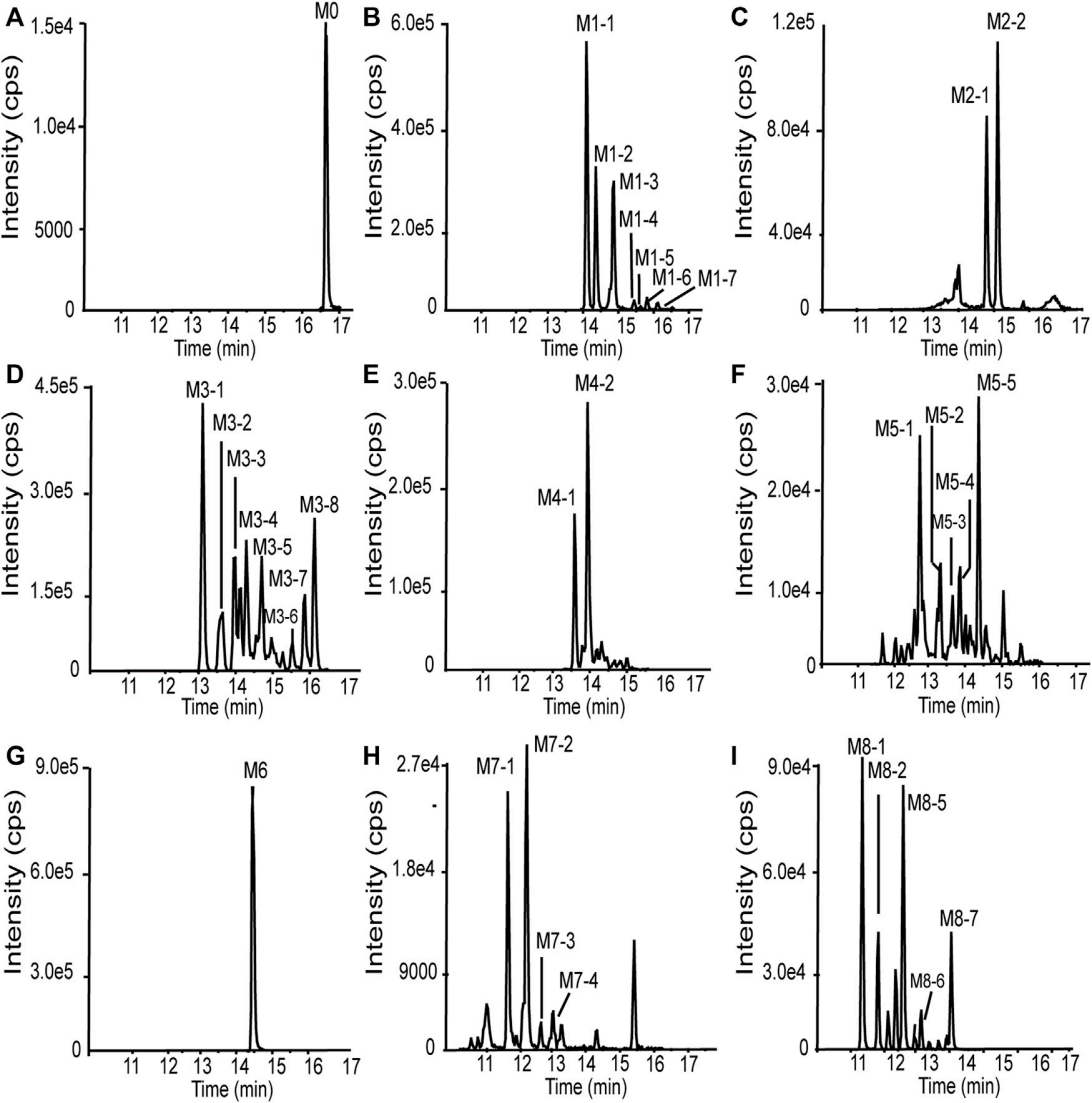
Extracted ion chromatograms of 101BHG-D01 and the metabolites in human liver microsomes. **(A)** M0 (101BHG-D01). **(B)** M1 metabolites. **(C)** M2 metabolites. **(D)** M3 metabolites. **(E)** M4 metabolites. **(F)** M5 metabolites. **(G)** M6 metabolite. **(H)** M7 metabolites. **(I)** M8 metabolites.

**FIGURE 4:**
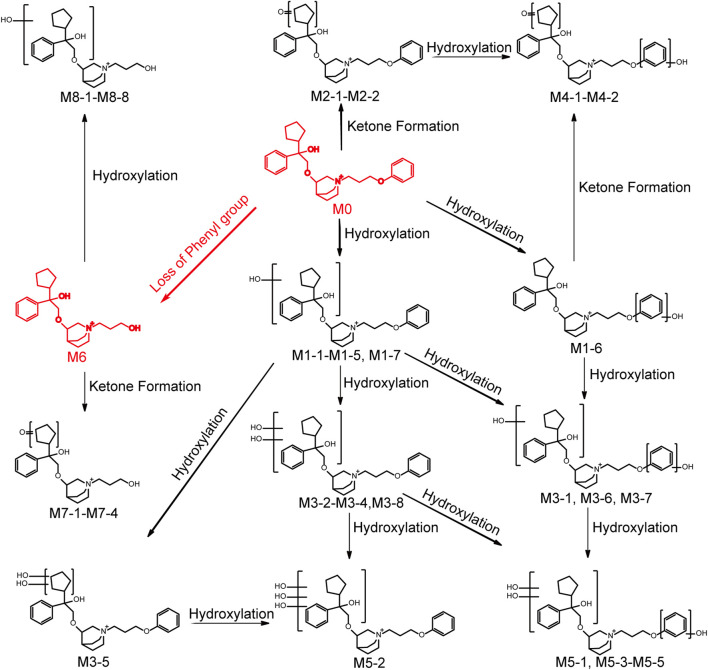
Chemical structures of the metabolites and proposed metabolic pathways of 101BHG-D01 in human liver microsomes.

101BHG-D01 and its 36 metabolites were identified in human liver microsomes by using HPLC-Q/TOF-MS method. According to the MS/MS information, the characteristic metabolic pathways involved hydroxylation, ketone formation and loss of phenyl group. As shown in Figure 1, we divided the structure of 101BHG-D01 into 2 parts, Part A and Part B, to characterize the structures of metabolites clearly. The detailed information, XIC, structures of the metabolites and the proposed metabolic pathways are shown in Table 3 and Figures 3,4.

**TABLE 3:** Summary of 101BHG-D01 and its metabolites *in vitro* and *in vivo*.

Metabolite id	Metabolite description	Formula	m/z	Error (ppm)	t_R_ (min)	Relative amounts *in vitro* (%)^a^	Relative amounts *in vivo* (%)^a^ ^,^ ^b^		
							Plasma	Urine	Feces
M0	Parent	C_29_H_40_NO_3_	450.3005	0.5	16.83	0.39	90.9 ± 3.2	73.4 ± 7.3	75.0 ± 9.3
M1-1	Hydroxylation	C_29_H_40_NO_4_	466.2952	0.0	14.38	11.56			
M1-2	Hydroxylation	C_29_H_40_NO_4_	466.2944	−1.7	14.58	6.07			
M1-3	Hydroxylation	C_29_H_40_NO_4_	466.2949	−0.6	14.96	7.25			
M1-4	Hydroxylation	C_29_H_40_NO_4_	466.2954	0.4	15.41	0.40	3.3 ± 1.6	3.1 ± 0.8	21.7 ± 8.9
M1-5	Hydroxylation	C_29_H_40_NO_4_	466.2948	−0.9	15.55	0.12			
M1-6	Hydroxylation	C_29_H_40_NO_4_	466.2947	−1.1	15.70	0.46			
M1-7	Hydroxylation	C_29_H_40_NO_4_	466.2954	0.4	15.92	0.30			
M2-1	Ketone Formation	C_29_H_38_NO_4_	464.2792	−0.6	14.85	1.59	-	0.4 ± 0.2	0.5 ± 0.2
M2-2	Ketone Formation	C_29_H_38_NO_4_	464.2795	0.0	15.09	2.22			
M3-1	Di-Hydroxylation	C_29_H_40_NO_5_	482.2893	−1.7	13.01	7.49			
M3-2	Di-Hydroxylation	C_29_H_40_NO_5_	482.2894	−1.5	13.31	3.02			
M3-3	Di-Hydroxylation	C_29_H_40_NO_5_	482.2896	−1.0	13.54	3.86			
M3-4	Di-Hydroxylation	C_29_H_40_NO_5_	482.2901	0.0	13.74	4.33	-	2.4 ± 0.8	1.5 ± 0.6
M3-5	Di-Hydroxylation	C_29_H_40_NO_5_	482.2899	−0.4	13.99	5.23			
M3-6	Di-Hydroxylation	C_29_H_40_NO_5_	482.2902	0.2	14.50	1.07			
M3-7	Di-Hydroxylation	C_29_H_40_NO_5_	482.2894	−1.5	14.71	2.65			
M3-8	Di-Hydroxylation	C_29_H_40_NO_5_	482.2901	0.0	14.87	4.64			
M4-1	Hydroxylation + Ketone Formation	C_29_H_38_NO_5_	480.2742	−0.4	13.56	3.63	-	1.7 ± 0.6	-
M4-2	Hydroxylation + Ketone Formation	C_29_H_38_NO_5_	480.2742	−0.4	13.84	7.05			
M5-1	Tri-Hydroxylation	C_29_H_40_NO_6_	498.2848	−0.6	12.51	0.62	-	-	-
M5-2	Tri-Hydroxylation	C_29_H_40_NO_6_	498.2850	0.0	12.95	0.35			
M5-3	Tri-Hydroxylation	C_29_H_40_NO_6_	498.2849	−0.2	13.22	0.20			
M5-4	Tri-Hydroxylation	C_29_H_40_NO_6_	498.2849	−0.2	13.38	0.25			
M5-5	Tri-Hydroxylation	C_29_H_40_NO_6_	498.2850	0.0	13.79	0.49			
M6	Loss of Phenyl group	C_23_H_36_NO_3_	374.2686	−1.6	14.46	17.97	5.1 ± 2.0	8.1 ± 2.5	1.3 ± 0.6
M7-1	Loss of Phenyl group + Ketone Formation	C_23_H_34_NO_4_	388.2480	−0.5	12.00	0.41		
M7-2	Loss of Phenyl group + Ketone Formation	C_23_H_34_NO_4_	388.2478	−1.0	12.40	0.57			
M7-3	Loss of Phenyl group + Ketone Formation	C_23_H_34_NO_4_	388.2481	−0.3	12.71	0.05	-	6.1 ± 3.0	-
M7-4	Loss of Phenyl group + Ketone Formation	C_23_H_34_NO_4_	388.2485	0.8	12.97	0.08			
M8-1	Loss of Phenyl group + Hydroxylation	C_23_H_36_NO_4_	390.2633	−1.5	11.49	1.42			
M8-2	Loss of Phenyl group + Hydroxylation	C_23_H_36_NO_4_	390.2637	−0.5	11.84	0.57			
M8-3	Loss of Phenyl group + Hydroxylation	C_23_H_36_NO_4_	390.2639	0.0	12.07	0.18			
M8-4	Loss of Phenyl group + Hydroxylation	C_23_H_36_NO_4_	390.2637	−0.5	12.24	0.42	0.7 ± 1.0	5.0 ± 2.4	-
M8-5	Loss of Phenyl group + Hydroxylation	C_23_H_36_NO_4_	390.2633	−1.5	12.40	1.38			
M8-6	Loss of Phenyl group + Hydroxylation	C_23_H_36_NO_4_	390.2637	−0.5	12.79	0.41			
M8-7	Loss of Phenyl group + Hydroxylation	C_23_H_36_NO_4_	390.2637	−0.5	13.46	1.32			

^a^ Relative amounts *in vitro* or *in vivo* (%) = peak area of 101BHG-D01, and each metabolite/total peak area in HLM, human plasma, urine or feces, respectively.

^b^ The values of relative contents *in vivo* were mean ± SD.

HLM, human liver microsomes; t_R_, retention time; -, not detected.

#### 3.3.1 Mass spectra analysis of parent drug

Before characterization of 101BHG-D01 metabolites, the chromatographic and mass spectroscopic behavior of the parent drug was investigated. The characteristic product ions of the parent drug were the substructural template for interpreting the structure of the metabolites.

**FIGURE 5:**
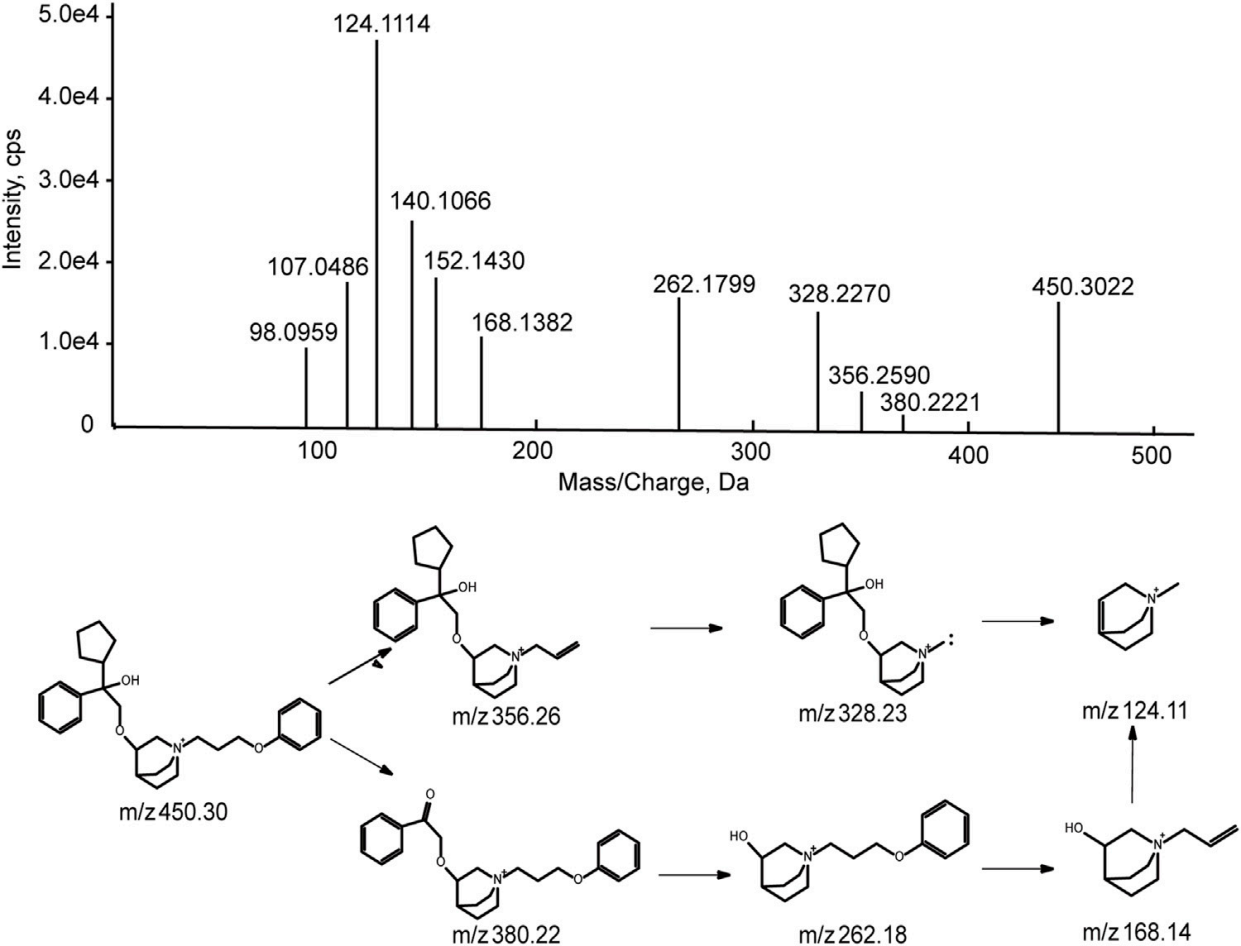
Accurate MS/MS spectra of 101BHG-D01 and its proposed fragmentation pathway.

101BHG-D01 was eluted at 16.8 min under the experimental condition. The accurate mass measurement gave the molecular ion at m/z 450.2985 with a theoretical elemental composition of C_29_H_40_NO_3_ in the positive mode. The main fragment ions were observed at m/z 356.26 (C_23_H_34_NO_2_^+^), 328.23 (C_21_H_30_NO_2_^+^), 262.18 (C_16_H_24_NO_2_^+^), 168.14 (C_10_H_18_NO^+^), and 124.11 (C_8_H_14_NO^+^), respectively, *via* the cleavage of carbon-carbon bond and carbon-oxygen bond. The proposed fragmentation pathway and MS/MS spectrum of 101BHG-D01 are shown in Figure 5.

#### 3.3.2 Identification of metabolites from 101BHG-D01

##### 3.3.2.1 Mono-hydroxylation (M1 metabolites)

Metabolites M1-1-M1-7 (t_R_ = 14.38, 14.58, 14.96, 15.41, 15.55, 15.70 and 15.92 min, respectively) gave the same molecular ions [M]^+^ at around m/z 466.295 (C_29_H_40_NO_4_) which were 16 Da (O) more than that of 101BHG-D01. In the MS/MS spectra of M1-1-M1-5 and M1-7, the fragment ions at m/z 372.25 and m/z 344.22 were also 16 Da heavier than those of the parent drug at m/z 356.26 and 328.23, respectively, and the fragment ions at m/z 262.18, 168.14, 152.14 and 124.11 were the same as those generated by the parent drug. Accordingly, M1-1-M1-5 and M1-7 were determined as mono-hydroxylated metabolites and hydroxylation might occur at the cyclopentyl group or phenyl group in the part A. In the MS/MS spectra of M1-6, the typical fragment ions at m/z 278.18, which was 16 Da higher than that of 101BHG-D01 at 262.18, and m/z 328.23, 168.14, 152.14 and 124.11, which were the same as those generated by the parent drug, indicated that hydroxylation might occur at the phenyl group in the part B.

##### 3.3.2.2 Mono-ketone formation (M2 metabolites)

Metabolites M2-1-M2-1 (t_R_ = 14.85 and 15.09 min) exhibited the same molecular ion [M]^+^ at m/z 464.280 (C_29_H_38_NO_4_) corresponding to the loss of a hydrogen molecule from the mono hydroxylated product of 101BHG-D01. Similar to the parent drug, the fragment ions at m/z 262.18, 168.14 and 124.11 were observed in their MS/MS spectra. The product ion at m/z 342.21 indicated that ketone formation had occurred at the cyclopentyl group in the part A.

##### 3.3.2.3 Di-hydroxylation (M3 metabolites)

Metabolites M3-1-M3-8 (t_R_ = 13.01, 13.31, 13.54, 13.74, 13.99, 14.50, 14.71 and 14.87 min, respectively) displayed the same molecular ions [M]^+^ at around m/z 482.290 (C_29_H_40_NO_5_). An increase of 32 Da in molecular weight compared to [M]^+^ ion of the parent drug suggested that M3 metabolites were the di-hydroxylated metabolites. In the MS/MS spectra of M3-1, M3-6 and M3-7, the ions at m/z 372.25 and 344.22 originated from the fragment ions of 101BHG-D01 at m/z 356.26 and 328.23, respectively, corresponding to the mono-hydroxylation in the part A. While the ion at 278.17, which was 16 Da heavier than that of the parent drug at m/z 262.18, indicated that the other hydroxylation might occur at the phenyl group in the part B. The main fragment ions at 388.25 and 360.22 in the MS/MS spectra of M3-2-M3-4 and M3-8, which were 32 Da higher than those of the parent drug at m/z 356.26 and 328.23, showed di-hydroxylation occurred in the part A. In the spectrum of M3-5, the characteristic ions at m/z 380.22, 262.18 and 124.11 were observed. Among them, the product ion with m/z 380.22 was formed by the loss of the cyclopentane part from the molecular cation, which suggested that the two hydroxyl groups were located at the cyclopentyl group.

##### 3.3.2.4 Hydroxylation and ketone formation (M4 metabolites)

Metabolite M4-1 and M4-2 (t_R_ = 13.56 and 13.84 min) were detected as molecular ions [M]^+^ at m/z 480.274 (C_29_H_38_NO_5_), which were 2 Da less than that of di-hydroxylated metabolites, indicating that they were the hydroxylated and ketone formed products of 101BHG-D01. The ketone formation occurred at cyclopentane because the key fragment ions were observed at m/z 370.24 and 342.21. The hydroxylation occurred at the phenyl group in the part B because the key fragment ion was observed at m/z 278.17.

##### 3.3.2.5 Tri-hydroxylation (M5 metabolites)

Metabolites M5-1-M5-5 (t_R_ = 12.51, 12.95, 13.22, 13.38 and 13.79 min, respectively) showed the same molecular ions [M]^+^ at around m/z 498.285 (C_29_H_40_NO_6_), which indicated an addition of 48 Da compared with the molecular ion of the parent drug. In the MS/MS spectra of M5-1, M5-3, M5-4 and M5-5, the characteristic fragment ions at m/z 388.25 and 360.22, which were 32 Da (2O) higher than those of the parent drug at m/z 356.26 and 328.23, indicated two hydroxyl groups were located at the cyclopentyl group or phenyl group in the part A. The fragment ion at m/z 278.18, which was 16 Da heavier than that of the parent drug at m/z 262.18, suggested that one hydroxyl group was located at the phenyl group in the part B. In the MS/MS spectrum of M5-2, the main fragment ions at m/z 376.21, 262.14, 168.14 and 124.11 showed that tri-hydroxylation occurred at the cyclopentyl group or phenyl group in the part A.

##### 3.3.2.6 Loss of phenyl group (M6 metabolite)

Metabolite M6 (t_R_ = 14.46 min) had a molecule ion [M]^+^ at m/z 374.269(C_23_H_36_NO_3_), which was 76 Da lower than that of the parent drug, corresponding to the loss of phenyl group compared with parent drug. In the MS/MS spectrum, the characteristic fragment ion at m/z 186.15 was also 76 Da lower than that of 101BHG-D01 at m/z 262.18, which indicated the loss of phenyl group occurred in the part B.

##### 3.3.2.7 Loss of phenyl group and ketone formation (M7 metabolites)

Metabolites M7-1-M7-4 (t_R_ = 12.00, 12.40, 12.71 and 12.97 min, respectively), with [M]^+^ ions at around m/z 388.248 (C_23_H_34_NO_4_), were 14 Da higher than that of M6 and the fragment ion at m/z 186.15 was similar to that of M6. Accordingly, M7 metabolites might be isomers of ketone formed product of M6 and ketone formation occurred at the cyclopentyl group in part A.

##### 3.3.2.8 Loss of phenyl group and hydroxylation (M8 metabolites)

Metabolites M8-1-M8-8 (t_R_ = 11.49, 11.84, 12.07, 12.24, 12.40, 12.79 and 13.46 min, respectively) generated the molecular ions [M]^+^ at around m/z 390.264 (C_23_H_36_NO_4_), which were 16 Da more than that of M6. The key product ion at m/z 186.15 was detected in their MS/MS spectra, which was the same as that of M6. Accordingly, M8 was identified as the hydroxylated product of M6 and the hydroxyl group was located at the cyclopentyl group or phenyl group in the part A.

### 3.4 Relative quantification of 101BHG-D01 and metabolites in human plasma, urine and feces

Based on the result of metabolite profiling in human liver microsomes, 9 ion pairs were chosen. The metabolites detected in human plasma, urine and feces and their relative amounts are listed in Table 3. By comparing the retention time of each peak in XIC of the dosing samples with that of the incubation sample, the parent drug, M1 metabolites, M6 and M8 metabolites were found in the plasma samples. The parent drug, M1-M4 and M6-M8 metabolites were all detected in the urine samples. The parent drug, M1-M3 and M6 metabolites were found in the fecal samples. The proposed metabolic pathways of 101BHG-D01 *in vivo* mainly included loss of phenyl group and hydroxylation. The semi-quantification result in circulation showed 101BHG-D01 represented the main proportion of total drug-related exposure (90.9% ± 3.2% of the total peak area). The M1, M6 and M8 metabolites accounted for 3.3 ± 1.6%, 5.1 ± 2.0%, and 0.7 ± 1.0% of the total drug-related exposure, which showed that no peak ratio of any metabolite exceeded 10%.

### 3.5 Safety and tolerability

Overall, 25 subjects (46.3%) reported 46 AEs during the study. The details of AEs in subjects who received the study drug and placebo in the study are summarized in Supplementary Material. Among the 46 AEs, the 40 AEs (38.9%) were considered by the investigators as possibly related to the investigational drugs (101BHG-D01 inhalation and placebo) and reported as adverse drug reactions. The incidence of adverse drug reactions in the 101BHG-D01 inhalation treatment was lower than that in the placebo treatment (60%), and there was no obvious dose correlation. The most frequently adverse drug reactions were abnormal ECG and laboratory results, including: hyponatremia, hypertriglyceridemia, increased alanine aminotransferase, increased total bile acid, and sinus bradycardia. Oral ulcer and palpitation, which were considered to be related to the pharmacological effects of anticholinergics, had low incidence. All the adverse drug reactions were mild in intensity and reported as grade 1 (U.S. Department of Health and Human Services, 2017). The investigators confirmed that the adverse drug reactions had no clinical significance. No subject withdrew from the study due to the adverse events. No serious adverse reactions occurred. All events were resolved at the end of the trial. In general, 101BHG-D01 was safe and well tolerated in healthy subjects.

## 4 Discussion

This is the first clinical trial to investigate the pharmacokinetics, metabolite profiling, safety and tolerability of 101BHG-D01 delivered by MDI after single inhalation in Chinese healthy subjects. Meanwhile, according to the results of the metabolite profiling *in vitro*, the main metabolite of 101BHG-D01 was identified as M6, which corresponding to the loss of phenyl group compared with the parent drug. The pharmacokinetic profiles of M6 were also investigated. 101BHG-D01 was safe and well tolerated in this study.

In terms of the pharmacokinetic study, following single inhalation, 101BHG-D01 was rapidly absorbed into plasma and eliminated slowly. The T_max_ over the dose range was about 5 min, and the mean values of t_1/2z_ were about 30 h. 101BHG-D01 inhalation aerosol is indicated for the treatment of COPD, which requires the study drug helps patients relieve the symptoms quickly. The immediate absorption showed 101BHG-D01 could reach its peak concentration in a few minutes, and implied 101BHG-D01 inhalation aerosol could exhibit its therapeutic effect rapidly after administration. The half-life of 101BHG-D01 is longer than that of bencycloquidium bromide (BCQB, about 15 h) (Luo et al., 2021), which indicates 101BHG-D01 has a higher affinity to the M receptors. BCQB is another M receptor antagonist. Its nasal spray has been approved by Chinese National Medical Products Administration (NMPA) and its inhalation aerosol is under the clinical trial. The long half-life of 101BHG-D01 suggests the frequency of clinical administration, and once-daily dosing can be explored in the following dose exploration studies in COPD patients. These pharmacokinetic profiles are similar to the other anticholinergic bronchodilators (Cahn et al., 2013; Algorta et al., 2016; Sechaud et al., 2016). The systemic exposure to 101BHG-D01 after inhaled administration was low, when measured by C_max_ and AUC parameters. For the conventional oral drugs, after absorbed into the circulation, drugs are transported to the target organs by bloodstream. Contrary to the conventional oral drugs, after inhalation, 101BHG-D01 directed to its target organ lung, and then some of it was released from its target organ lung to blood, which resulted in its low systemic exposure. This characteristic might guarantee the safety of the study drug and minimize cardiovascular side effects resulted from the undesired binding with other muscarinic receptors in heart. M6 reached its peak concentration later than the parent drug and eliminated more rapidly than the parent drug. The C_max_ and AUC of 101BHG-D01 increased with dose escalation in the range of 20 to 600 μg, but the increasing rates were higher than the dose increasing rate. Compared with the 600 μg dose cohort, the significant increase of the systemic exposure to 101BHG-D01 in the 900 μg dose cohort was not observed. The C_max_ and AUC values of 101BHG-D01 increased out of proportion to the whole studied doses, which meant the linear pharmacokinetic characteristics of 101BHG-D01 was not observed. These results might be related to the large inter-individual variability of the C_max_ and AUC of 101BHG-D01.101BHG-D01 is a substrate for CYP2D6, and CYP2D6 is a drug-metabolizing enzyme with gene polymorphism. This characteristic might explain the large inter-subject variability of the PK parameters. Hence, the CYP2D6 genotyping in patients need to be considered in the Phase II and III studies. On the other hand, with the inhaled doses ascending, the binding sites of the M receptors might be saturated with the study drug, which might also result in that the C_max_ and AUC values of 101BHG-D01 increased out of proportion to the whole studied doses. The cumulative excretion rates of 101BHG-D01 and M6 in urine were low, which indicated that the renal excretion might not be a main elimination route for the study drug. This characteristic indicates that the treatment of COPD with 101BHG-D01 might reduce the risk of urinary retention. If the study drug is mainly excreted in urine, it would bind with the muscarinic M3 receptors in detrusor smooth muscle, cause the detrusor smooth muscle relaxation and increase the risk of urinary retention. Urinary retention is an adverse drug reaction of tiotropium, and this undesirable safety profile limits its clinical application in patients with urinary retention. Fecal excretion might be the major excretion pathway of 101BHG-D01. These data support the progression of 101BHG-D01 inhalation aerosol in the Phase Ⅱ and III clinical study in patients with COPD.

The *in vitro* metabolic profiles of 101BHG-D01 and relative quantification of the parent drug and the metabolites *in vivo* were also thoroughly investigated using HPLC-Q/TOF-MS and LC-MS/MS method in this study. *In vitro*, 101BHG-D01 was incubated with human liver microsomes and 36 metabolites were identified. According to the unpublished data provided by Beijing Shuobai Pharmaceutical Technology Co., Ltd., compared with the metabolites identified in animal (mice, rat, beagle dog, and Macaca fascicularis) liver microsomes, there was no obvious difference from those identified in human liver microsomes, which demonstrated the nonclinical safety test has its reference value. Using the *in vitro* results as a reference, the relative contents of 101BHG-D01 and its metabolites in human plasma, urine and feces were calculated. The types and relative contents of the metabolites in plasma, urine and feces were different. The main characteristic metabolic bio-transformation routes involved hydroxylation and loss of phenyl group. No major metabolite that exceeded 10% of total drug-related systemic exposure was observed in the single-dose escalation study. In the subsequent multiple-dose escalation study, the relative quantification of the parent drug and metabolites was also conducted in the multiple administration dose (MAD) samples. The results show that the types and relative contents of the metabolites in the MAD samples are similar with those in the single administration dose (SAD) samples. The safety testing of drug metabolites guidance for industry of FDA emphasizes human metabolites that present at greater than 10 percent of total drug-related exposure at steady state can raise a safety concern. The above results indicate that the additional safety and toxicity assessments of the major metabolites are not necessary at present.

## 5 Conclusion

In conclusion, the initial pharmacokinetics, metabolite profiling, safety and tolerability of 101BHG-D01 indicate that it is a good candidate for the treatment of COPD and enable further clinical development in subsequent studies in COPD patients.

## Data Availability

The original contributions presented in the study are included in the article/Supplementary Material, further inquiries can be directed to the corresponding authors.
