# Supplementary material for: Pharmacokinetics, metabolite profiling, safety, and tolerability of inhalation aerosol of 101BHG-D01, a novel, long-acting and selective muscarinic receptor antagonist, in healthy Chinese subjects
Source: Front Pharmacol. 2022 Dec 15;13:1064364. doi: 10.3389/fphar.2022.1064364 (PMC9797597; doi:10.3389/fphar.2022.1064364)
Supplement: Supplementary file 1 [file Table1.DOCX]

**Supplementary Table 1 Number of adverse events and percentage of healthy subjects in the study.**

| Adverse events | 20 μg (n=4) | 60 μg (n=8) | 150 μg (n=8) | 300 μg (n=8) | 600 μg (n=8) | 900 μg (n=8) | Placebo (n=10) | Total (n=54) |
| --- | --- | --- | --- | --- | --- | --- | --- | --- |
|  | n (%) | n (%) | n (%) | n (%) | n (%) | n (%) | n (%) | n (%) |
| Hyponatremia | 0(0) | 0(0) | 2(25.0) | 0(0) | 0(0) | 0(0) | 0(0) | 2(3.7) |
| Hypertriglyceridemia | 1(25.0) | 0(0) | 0(0) | 0(0) | 1(12.5) | 0(0) | 0(0) | 2(3.7) |
| Hyperuricemia | 0(0) | 1(12.5) | 0(0) | 0(0) | 0(0) | 0(0) | 0(0) | 1(1.9) |
| Dizziness | 0(0) | 1(12.5) | 0(0) | 0(0) | 0(0) | 0(0) | 0(0) | 1(1.9) |
| Conjunctivitis | 0(0) | 0(0) | 1(12.5) | 0(0) | 0(0) | 0(0) | 0(0) | 1(1.9) |
| Elevated γ-glutamyltransferase | 0(0) | 0(0) | 0(0) | 0(0) | 0(0) | 0(0) | 1(10.0) | 1(1.9) |
| Decreased white blood cell count | 0(0) | 0(0) | 1(12.5) | 0(0) | 0(0) | 1(12.5) | 0(0) | 2(3.7) |
| Elevated alanine aminotransferase | 0(0) | 0(0) | 0(0) | 0(0) | 0(0) | 2(25.0) | 1(10.0) | 3(5.6) |
| Elevated low density lipoprotein | 0(0) | 0(0) | 1(12.5) | 0(0) | 0(0) | 0(0) | 0(0) | 1(1.9) |
| Elevated amylase | 0(0) | 0(0) | 0(0) | 1(12.5) | 0(0) | 0(0) | 0(0) | 1(1.9) |
| Drop in red blood cell count | 0(0) | 0(0) | 1(12.5) | 0(0) | 0(0) | 0(0) | 0(0) | 1(1.9) |
| Decreased hematocrit | 0(0) | 0(0) | 1(12.5) | 0(0) | 0(0) | 0(0) | 0(0) | 1(1.9) |
| Decreased percentage of lymphocytes | 0(0) | 0(0) | 1(12.5) | 0(0) | 0(0) | 0(0) | 0(0) | 1(1.9) |
| Decreased lymphocyte count | 0(0) | 0(0) | 0(0) | 0(0) | 0(0) | 1(12.5) | 0(0) | 1(1.9) |
| Urine white blood cell positive | 0(0) | 0(0) | 0(0) | 0(0) | 0(0) | 0(0) | 1(10.0) | 1(1.9) |
| ECG T wave abnormality | 0(0) | 0(0) | 0(0) | 0(0) | 1(12.5) | 0(0) | 0(0) | 1(1.9) |
| Elevated blood cholesterol | 0(0) | 0(0) | 1(12.5) | 0(0) | 0(0) | 0(0) | 0(0) | 1(1.9) |
| Elevated blood triglycerides | 0(0) | 0(0) | 0(0) | 1(12.5) | 0(0) | 0(0) | 1(10.0) | 2(3.7) |
| Decreased hemoglobin | 0(0) | 0(0) | 1(12.5) | 0(0) | 0(0) | 0(0) | 0(0) | 1(1.9) |
| Elevated blood creatine phosphokinase | 0(0) | 0(0) | 0(0) | 0(0) | 0(0) | 0(0) | 1(10.0) | 1(1.9) |
| Decreased blood phosphorus | 0(0) | 0(0) | 0(0) | 1(12.5) | 0(0) | 0(0) | 0(0) | 1(1.9) |
| Lower blood pressure | 0(0) | 0(0) | 0(0) | 0(0) | 1(12.5) | 0(0) | 0(0) | 1(1.9) |
| Increased percentage of neutrophils | 0(0) | 0(0) | 1(12.5) | 1(12.5) | 0(0) | 0(0) | 0(0) | 2(3.7) |
| Decreased neutrophil count | 0(0) | 0(0) | 1(12.5) | 0(0) | 0(0) | 0(0) | 0(0) | 1(1.9) |
| Elevated neutrophil count | 0(0) | 0(0) | 0(0) | 1(12.5) | 0(0) | 0(0) | 0(0) | 1(1.9) |
| Increased total bile acids | 0(0) | 0(0) | 1(12.5) | 1(12.5) | 0(0) | 0(0) | 1(10.0) | 3(5.6) |
| Syncope | 0(0) | 0(0) | 0(0) | 1(12.5) | 0(0) | 0(0) | 1(10.0) | 2(3.7) |
| Stuffy nose | 0(0) | 0(0) | 1(12.5) | 0(0) | 0(0) | 0(0) | 0(0) | 1(1.9) |
| Uterine adnexal cyst | 0(0) | 0(0) | 0(0) | 0(0) | 0(0) | 0(0) | 1(10.0) | 1(1.9) |
| Mouth ulcers | 0(0) | 0(0) | 1(12.5) | 0(0) | 0(0) | 0(0) | 0(0) | 1(1.9) |
| Palpitations | 0(0) | 0(0) | 0(0) | 0(0) | 0(0) | 1(12.5) | 1(10.0) | 2(3.7) |
| Sinus bradycardia | 0(0) | 0(0) | 0(0) | 0(0) | 1(12.5) | 0(0) | 2(20.0) | 3(5.6) |

n (%) = number and percentage of healthy subjects of each group in the respective adverse event categories.
